# Supplementary material for: Economic burden of pulmonary arterial hypertension in Switzerland
Source: PLoS One. 2026 Apr 28;21(4):e0348190. doi: 10.1371/journal.pone.0348190 (PMC13123976; doi:10.1371/journal.pone.0348190)

**Economic Burden of Pulmonary Arterial Hypertension in Switzerland.**

**Supplementary materials**

**Tables**

[Table S1. Unit costs used for the direct cost calculation (in EUR). 2](#_Toc225260948)

[Table S2. Unit costs used for the indirect cost calculation (in EUR). 4](#_Toc225260949)

[Table S3. Healthcare resource use according to medical record data and patient survey. 5](#_Toc225260950)

[Table S4. Estimated direct and indirect costs per patient per year (EUR). 7](#_Toc225260951)

[Table S5. Estimated total costs (EUR) by WHO functional class and ESC/ERS risk – details on data dispersion 8](#_Toc225260952)

[Table S6. Estimated costs (EUR) by years since diagnosis. 9](#_Toc225260953)

[Table S7. Results of the generalized linear regression model (with total costs as dependent variable). 10](#_Toc225260954)

[Table S8. Results of the generalized linear regression model including PAH subtype, WHO-FC, and ESC/ERS Risk. 11](#_Toc225260955)

[Table S9. Results of the sensitivity analysis in which 4-week and 6 month outcomes were annualized using lower or higher multiplication factors. 12](#_Toc225260956)

**Figures**

[Figure S1. Estimated total costs per patients per year, by gender (EUR). 13](#_Toc225260957)

[Figure S2. Estimated direct costs per patients per year, by gender (EUR). 13](#_Toc225260958)

[Figure S3. Estimated indirect costs per patients per year, by gender (EUR). 14](#_Toc225260959)

[Figure S4. Estimated total costs per patients per year, by age group (EUR). 14](#_Toc225260960)

[Figure S5. Estimated direct costs per patients per year, by age group (EUR). 15](#_Toc225260961)

[Figure S6. Estimated indirect costs per patients per year, by age group (EUR). 15](#_Toc225260962)

[Figure S7. Estimated total costs per patients per year, by gender and WHO functional class (EUR). 16](#_Toc225260963)

[Figure S8. Estimated total costs per patients per year, by gender and ESC/ERS Risk (EUR). 16](#_Toc225260964)

[Figure S9. Estimated total costs per patients per year, by age group and WHO functional class 17](#_Toc225260965)

[Figure S10. Estimated total costs per patients per year, by age group and ESC/ERS risk (EUR). 17](#_Toc225260966)

[Figure S11. Estimated total costs per patients per year, by years since PAH diagnosis (EUR). 18](#_Toc225260967)

[Figure S12. Estimated total costs per patients per year, by PAH subtype (EUR). 18](#_Toc225260968)

[Figure S13. Estimated total costs per patients per year, by number of co-morbidities (EUR). 19](#_Toc225260969)

[Figure S14. Estimated total costs per patients per year, by number of prescriber PAH drugs (EUR). 19](#_Toc225260970)

[Figure S15. Estimated total costs per patients per year, by gender and years since PAH diagnosis (EUR). 20](#_Toc225260971)

Table S1. Unit costs used for the direct cost calculation (in EUR).

| Direct costs | EUR | Sources and comments |
| --- | --- | --- |
| Hospitalisation costs per day |  | Federal Office of Public Health (FOPH). Auswirkungen der Covid-19-Pandemie auf die Kostenträger im Gesundheitswesen. Zweiter Zwischenbericht des Bundesrates in Erfüllung des Postulates 20.3135 SGK-SR vom 21. April 2020. Accessed June 26, 2024.  Estimates from a report commissioned by the Swiss Confederation—based on data from four major health insurers and focusing on COVID-19 hospitalizations—were used as a proxy. For intermediate care stays, a midpoint value between general ward and ICU costs was assumed. |
| ICU | 4,188.37 |  |
| Intermediate care | 2,967.46 |  |
| General ward | 1,745.50 |  |
| Costs per outpatient visit | 145.39 | TARMED: Online-Tarifbrowser. Accessed June 12, 2025. <https://browser.tartools.ch/de/tarmed_kvg>  Assuming a 30-minute consultation and including the writing of a short report. Tarmed codes 00.0010, 00.0020, 00.030, and 00.2206 were used. |
| Costs per emergency visit | 479.57 | Swiss Health Observatory - Bulletin 10/2024: Ambulante Konsultationen in Notfallstationen. Accessed June 12, 2025. <https://www.obsan.admin.ch/de/publikationen/2024-ambulante-konsultationen-notfallstationen> |
| Diagnostic costs |  | TARMED: Online-Tarifbrowser. Accessed June 12, 2025. <https://browser.tartools.ch/de/tarmed_kvg> |
| *6-MWD* | 45.00 | Tarmed code 15.0300 |
| *CPET* | 323.78 | Tarmed code 15.0320 |
| *BNP or NT-proBNP* | 65.97 | Analysis list:  <https://www.bag.admin.ch/bag/de/home/versicherungen/krankenversicherung/krankenversicherung-leistungen-tarife/Analysenliste.html>  Code 1576.00 / 1576.01 |
| *ECG* | 35.53 | Tarmed code 17.0010 |
| *Echocardiography* | 364.69 | Tarmed code 17.0210 |
| *Cardiac MRI* | 521.14 | Tarmed codes 39.5100 and 17.0410  The evaluation of the test results by a cardiologist was included in the costs. |
| *Arterial blood gas or pulse oximetry* | 111.04 | Tarmed code 15.0710 |
| *Hemodynamics* | 47.33 | Tarmed code 17.0410 |
| *Pulmonary function test* | 129.02 | Tarmed code 15.0160 |
| *Right heart catheterization* | 529.83 | Tarmed code 17.0910 |
| *CT* | 224.75 | Tarmed code 39.4060 |
| *Blood or urine test* | 209.42 | The number of tests (or investigated substances) may vary considerably between PAH patient and treating centre.  For the present analyses, a conservative flat-rate of CHF200 was assumed. |
| *Chest x-ray* | 33.60 | Tarmed code 39.0190 |
| *Sonography* | 49.76 | Tarmed code 39.3510 |
| PAH treatment costs |  |  |
| Oxygen therapy (per day) | 23.84 | Mittel und Gegenständeliste (MiGeL). Accessed June 12, 2025. <https://www.bag.admin.ch/bag/de/home/versicherungen/krankenversicherung/krankenversicherung-leistungen-tarife/Mittel-und-Gegenstaendeliste.html>  The costs included equipment rental (stationary and portable), oxygen refills, maintenance, and consumables.  MiGel positions-Nr. 14.10.20.00.2, 14.10.51.00.2, 14.10.22.00.2, 14.10.52.00.2, 14.10.50.01.1, and 14.10.60.00.1 were included. |
| Drug therapy (per tablet/vial) |  | Specialty list. Accessed June 12, 2025. <https://www.xn--spezialittenliste-yqb.ch/> |
| *Macicentan (10mg)* | 106.80 | Based on a 30-unit package.  Price per package: CHF3,062.65. |
| *Tadalafil (20mg)* | 9.42 | Based on a 56-unit package.  Price per package: CHF511.30. |
| *Riociguat (1.5-2.0mg)* | 35.60 | Based on a 84-unit package.  Price per package: CHF2,819.45. |
| *Treprostinil (20mg/ml)* | 2,755.95 | Based on a 20mg/ml vial.  Price per vial: CHF2,631.85 |
| *Sildenafil (20mg)* | 6.28 | Based on a 90-unit package.  Price per package: CHF522.10. |
| *Selexipag (200-1000mcg)* | 84.81 | Based on a 60-unit package (the price is the same for all dosages).  Price per package: CHF4854.30. |
| *Ambrisentan (5-10mg)* | 41.88 | Based on a 30-unit package.  Price per package: CHF1,195.35. |
| *Bosentan (62.5-125mg)* | 16.75 | Based on a 56-unit package (the price is the same for all dosages)  Price per package: CHF922.05. |
| *Torasemid (2.5-15mg)* | 0.15-0.29 | Based on a 100-unit packages. Price depends on the dosage.  Price per package (2.5mg): CHF13.5.  Price per package (5mg): CHF15.58.  Price per package (10mg): CHF27.55.  Price per package (200mg): CHF70.50. |
| *Amlodipin (10mg)* | 0.61 | Based on a 100-unit package (dosage 10mg).  Price per package: CHF58.10. |
| Familiy doctor visits | 106.42 | Assuming a 30-minute consultation. Tarmed codes 00.0010, 00.0020, 00.030 were used |
| Specialist visits | 157.06 | H+. Der neue Tarif für die psychologische Psychotherapie gilt definitiv ab dem 1. Januar 2023. Accessed June 12, 2025. <https://www.hplus.ch/de/publikationen/eflash/ausgaben/11/2022/der-neue-tarif-fuer-die-psychologische-psychotherapie-gilt-definitiv-ab-dem-1-januar-2023>  Tarif des consultations diététiques. Accessed June 12, 2025. <https://feedgood.ch/tarifs-remboursement/> Accessed June 12, 2025. |

Note: Swiss Francs (CHF) were converted to Euros using an average exchange rate of CHF1.000=EUR1.047 for the year 2024.

Table S2. Unit costs used for the indirect cost calculation (in EUR).

| Earning per hour | Females | Males | Both | Sources and comments |
| --- | --- | --- | --- | --- |
| Total | 37.59 | 42.15 | 40.34 | Swiss Federal Statistical Office - Wages, income from employment and labour costs. Accessed June 12, 2025. <https://www.bfs.admin.ch/bfs/en/home/statistics/work-income/wages-income-employment-labour-costs.html> |
| Under 20 years | 26.34 | 26.89 | 26.60 |  |
| 20 - 29 years | 31.42 | 32.55 | 32.08 |  |
| 30 - 39 years | 39.31 | 40.93 | 40.34 |  |
| 40 - 49 years | 40.96 | 46.51 | 44.25 |  |
| 50 - 64/65 years | 40.56 | 48.17 | 45.00 |  |
| Over 64/65 years | 35.51 | 43.03 | 39.68 |  |
|  |  |  |  |  |
| Costs of professional home care services per hour | 55.08 |  |  | Spitex - Kassenpflichtige Leistungen. Accessed June 12, 2025. <https://www.spitex.ch/Spitex/Tarife/Kassenpflichtige-Leistungen/PaAVw/> |

Note: Swiss Francs (CHF) were converted to Euros using an average exchange rate of CHF1.000=EUR1.047 for the year 2024.

Table S3. Healthcare resource use according to medical record data and patient survey.

| **Parameter, mean ± SD or n (%)** |  |  |  |  |  |  |
| --- | --- | --- | --- | --- | --- | --- |
| **All PAH patients (N=124)** | **Mean** | **Standard deviation or %** | **Median** | **Minimum** | **Maximum** | **95% Confidence interval** |
| Number of hospitalisations (over 6 months) | 0.29 | 0.719 | 0 | 0 | 4 | 0.16-0.42 |
| Length of stay (days over 6 months) | 2.55 | 10.30 | 0 | 0 | 100 | 0.72-4.38 |
| *ICU* | 0 | 0 | 0 | 0 | 0 | - |
| *Intermediate care* | 0.14 | 0.92 | 0 | 0 | 9 | -0.03-0.30 |
| *Normal ward* | 2.43 | 9.61 | 0 | 0 | 91 | 0.72-4.14 |
| Number of outpatient visits to PAH centre (over 6 months ) | 5.13 | 5.34 | 3 | 0 | 34 | 4.18-6.08 |
| Diagnostic tests (over 6 months) |  |  |  |  |  |  |
| *6MWD* | 1.07 | 0.94 | 1 | 0 | 6 | 0.90-1.24 |
| *CPET* | 0.19 | 0.45 | 0 | 0 | 2 | 0.11-0.27 |
| *BNP or NT-proBNP* | 1.59 | 1.41 | 1 | 0 | 12 | 1.34-1.85 |
| *ECG* | 0.48 | 0.85 | 0 | 0 | 4 | 0.34-0.64 |
| *Echocardiography* | 0.78 | 0.67 | 1 | 0 | 3 | 0.66-0.91 |
| *cMRI* | 0.08 | 0.28 | 0 | 0 | 1 | 0.03-0.13 |
| *Arterial blood gas or pulse oxymetry* | 0.98 | 1.32 | 1 | 0 | 10 | 0.74-1.22 |
| *Hemodynamics (e.g. Doppler ulstasound)* | 0.55 | 1.20 | 0 | 0 | 8 | 0.31-0.67 |
| *Right Hearth Catherization* | 0.34 | 0.48 | 0 | 0 | 1 | 0.25-0.42 |
| *Pulmonary function test* | 0.86 | 0.76 | 1 | 0 | 3 | 0.72-1.00 |
| *CT scan* | 0.24 | 0.55 | 0 | 0 | 3 | 0.14-0.34 |
| *Blood/Urine test* | 0.70 | 1.47 | 0 | 0 | 9 | 0.45-0.98 |
| *Radiography* | 0.10 | 0.39 | 0 | 0 | 3 | 0.03-0.17 |
| *Sonography* | 0.10 | 0.35 | 0 | 0 | 2 | 0.04-0.16 |
| *Treprostinil pump filling* | 1.18 | 3.14 | 0 | 0 | 18 | 0.63-1.77 |
| Number of emergency visits (over 6 months) | 0.11 | 0.39 | 0 | 0 | 3 | 0.04-0.18 |
| Proportion of patients with PAH treatment (in the last 6 months) |  |  |  |  |  |  |
| *Longterm oxygen therapy* | 46 | 37.1% |  |  |  |  |
| *Macitentan* | 87 | 70.2% |  |  |  |  |
| *Tadalafil* | 57 | 46.0% |  |  |  |  |
| *Riociguat* | 21 | 16.9% |  |  |  |  |
| *Treprostinil* | 20 | 16.1% |  |  |  |  |
| *Sildenafil* | 17 | 13.7% |  |  |  |  |
| *Selexipag* | 14 | 11.3% |  |  |  |  |
| *Ambrisentan* | 10 | 8.1% |  |  |  |  |
| *Bosentan* | 9 | 7.3% |  |  |  |  |
| *Torasemid* | 6 | 4.8% |  |  |  |  |
| *Amlodipin* | 3 | 2.4% |  |  |  |  |
| Number of visits outside PAH centres (over 4 weeks) |  |  |  |  |  |  |
| Number of visits to family doctor | 0.59 | 0.92 | 0 | 0 | 4 | 0.43-0.76 |
| Number of visits to specialists | 1.51 | 2.86 | 0 | 0 | 16 | 1.00-2.02 |
| Productivity loss |  |  |  |  |  |  |
| Work hours lost (over 4 weeks) | 30.70 | 59.14 | 0 | 0 | 180 | 14.71-36.44 |
| Household chores, hours lost per week | 8.49 | 34.78 | 0 | 0 | 342 | 1.69-15.81 |
| Formal care hours per week | 0.67 | 1.91 | 0 | 0 | 10 | 0.33-1.14 |
| Informal care hours per week | 4.71 | 15.62 | 0 | 0 | 140 | 1.56-8.16 |
| Informal care by a caregiver below retirement age per week | 3.39 | 13.77 | 0 | 0 | 124 | 0.40-6.03 |

Table S4. Estimated direct and indirect costs per patient per year (EUR).

| N=124 | Mean | Standard deviation or % | Median | Minimum | Maximum | 95% Confidence interval (lower bound) | 95% Confidence interval (higher bound) |
| --- | --- | --- | --- | --- | --- | --- | --- |
| Diagnostic costs | 2,359 | 1,705 | 2,083 | 0 | 9,845 | 2,056 | 2,662 |
| Outpatient visit costs | 1,491 | 1,553 | 872 | 0 | 9,886 | 1,215 | 1,767 |
| Treatment costs | 90,205 | 117,611 | 47,038 | 0 | 585,439 | 69,299 | 111,111 |
| Hospitalization costs | 9,288 | 38,071 | 0 | 0 | 371,086 | 2,520 | 16,055 |
| GP visit costs | 814 | 1,272 | 0 | 0 | 5,534 | 588 | 1,041 |
| Specialist visits costs | 3,063 | 5,819 | 0 | 0 | 32,669 | 2,028 | 4,097 |
| Formal care costs | 1,894 | 5,425 | 0 | 0 | 28,640 | 930 | 2,859 |
| Total direct costs per year | 109,114 | 127,184 | 57,698 | 2,779 | 588,368 | 86,507 | 131,722 |
| Workdays lost costs | 16,944 | 32,940 | 0 | 0 | 112,709 | 11,089 | 22,799 |
| Household chores costs | 7,469 | 18,663 | 0 | 0 | 94,088 | 4,151 | 10,787 |
| Informal care costs | 5,431 | 18,252 | 0 | 0 | 117,483 | 2,186 | 8,675 |
| Total indirect costs per year | 29,844 | 48,200 | 2,519 | 0 | 228,450 | 21,276 | 38,413 |
| Total costs per year | 138,958 | 144,281 | 80,320 | 2,779 | 637,632 | 113,311 | 164,606 |

Note: Swiss Francs (CHF) were converted to Euros using an average exchange rate of CHF1.000=EUR1.047 for the year 2024.

Table S5. Estimated total costs (EUR) by WHO functional class and ESC/ERS risk – details on data dispersion

| **By WHO functional class** | Mean | SD | Median | IQR | Percentiles | | | Bootstrapped 96% CI | |
| --- | --- | --- | --- | --- | --- | --- | --- | --- | --- |
|  |  |  |  |  | 25 | 50 | 75 | Lower bound | Higher bound |
| **1 (N=26)** | 81,957 | 82,686 | 50,350 | 73,442 | 37,001 | 50,350 | 110,443 | 52,683 | 115,195 |
| **2 (N=52)** | 139,836 | 157,838 | 81,434 | 92,575 | 48,035 | 81,434 | 140,611 | 98,536 | 184,296 |
| **3 (N=37)** | 163,481 | 145,072 | 105,268 | 142,719 | 58,733 | 105,268 | 201,452 | 105,960 | 205,067 |
| **4 (N=5)** | 166,570 | 67,622 | 171,480 | 125,334 | 102,675 | 171,480 | 228,009 | 102,675 | 223,713 |
|  | | | | | | | | | |
| **By Risk stratification ESC/ERS** |  | | | | | | | | |
| **I (N=48)** | 130,009 | 134,109 | 90,109 | 98,417 | 47,617 | 90,109 | 146,035 | 95,353 | 170,803 |
| **II (N=43)** | 131,394 | 138,381 | 80,079 | 97,758 | 56,733 | 80,079 | 154,491 | 93,569 | 173,445 |
| **III (N=25)** | 127,730 | 142,521 | 64,667 | 99,813 | 47,293 | 64,667 | 147,107 | 76,641 | 188,851 |
| **IV (N=4)** | 291,728 | 167,874 | 228,009 | 284,731 | 181,222 | 228,009 | 465,953 | 184,470 | 447,431 |
| Abbreviations: CI: Confidence interval; ESC/ERS: European Society of Cardiology / European Respiratory Society; IQR: Interquartile range; SD: Standard deviation *I= low risk, II= intermediate-low risk, III= intermediate-high risk, IV= high risk. | | | | | | | | | |

Table S6. Estimated costs (EUR) by years since diagnosis.

| Years since PAH diagnosis | | Diagnostic costs per year (€) | Outpatient visits to PAH centre costs per year (€) | Treatment costs per year (€) | Hospitali-sation costs per year (€) | GP visits costs per year (€) | Specialist visits costs per year (€) | Formal care costs per year (€) | Direct costs per year (€) | Workdays lost costs per year (€) | Household chores costs per year (€) | Informal care costs per year (€) | Indirect costs per year (€) | Total costs per year (€) |
| --- | --- | --- | --- | --- | --- | --- | --- | --- | --- | --- | --- | --- | --- | --- |
| <1 year (N=29) | Mean | 3,437 | 1,935 | 54,373 | 5,381 | 1,002 | 3,028 | 1,876 | 71,031 | 15,945 | 7,781 | 1,772 | 25,498 | 96,530 |
|  | Std. Deviation | 2,455 | 2,006 | 93,590 | 14,505 | 1,564 | 4,487 | 5,729 | 94,978 | 33,080 | 18,052 | 5,698 | 38,365 | 103,227 |
| 2-5 years (N=39) | Mean | 2,103 | 1,364 | 101,139 | 11,001 | 780 | 3,508 | 1,469 | 121,365 | 8,929 | 9,066 | 7,934 | 25,929 | 147,294 |
|  | Std. Deviation | 1,345 | 1,230 | 140,360 | 27,518 | 1,090 | 6,690 | 3,709 | 145,663 | 25,311 | 20,783 | 22,388 | 53,062 | 170,221 |
| 6-10 years (N=22) | Mean | 2,101 | 1,639 | 108,143 | 635 | 566 | 2,692 | 3,645 | 119,420 | 15,183 | 3,007 | 4,482 | 22,672 | 142,092 |
|  | Std. Deviation | 1,255 | 1,716 | 120,945 | 2,977 | 1,256 | 4,937 | 8,205 | 127,512 | 32,080 | 5,434 | 11,978 | 33,153 | 137,644 |
| 11-15 years (N=19) | Mean | 2,088 | 1,255 | 90,969 | 26,697 | 510 | 2,364 | 452 | 124,334 | 31,661 | 4,565 | 4,858 | 41,084 | 165,419 |
|  | Std. Deviation | 1,363 | 1,473 | 109,022 | 86,257 | 1,053 | 5,195 | 1,971 | 146,793 | 41,008 | 8,640 | 15,653 | 52,196 | 166,228 |
| >15 years (N=13) | Mean | 1,742 | 1,074 | 115,535 | 3,491 | 1,277 | 3,455 | 881 | 127,455 | 19,964 | 7,690 | 323 | 27,976 | 155,432 |
|  | Std. Deviation | 737 | 1,103 | 100,351 | 8,786 | 1,320 | 8,476 | 3,177 | 105,515 | 36,486 | 24,291 | 1,164 | 39,684 | 122,592 |
| Total | Mean | 2,379 | 1,502 | 91,236 | 9,440 | 805 | 3,063 | 1,737 | 110,161 | 16,440 | 6,820 | 4,557 | 27,818 | 137,979 |
|  | Std. Deviation | 1,712 | 1,564 | 118,289 | 38,365 | 1,268 | 5,854 | 5,119 | 127,942 | 32,733 | 17,064 | 15,293 | 44,855 | 145,080 |

Table S7. Results of the generalized linear regression model (with total costs as dependent variable).

| Parameter | Coefficient | Std. Error | 95% Wald Confidence Interval | | Hypothesis Test | | |
| --- | --- | --- | --- | --- | --- | --- | --- |
|  |  |  | Lower | Upper | Wald Chi-Square | df | Sig. |
| (Intercept) | 12.903 | 0.5134 | 11.897 | 13.909 | 631.669 | 1 | <0.001 |
| Female | -0.492 | 0.1661 | -0.817 | -0.166 | 8.756 | 1 | 0.003 |
| Age | -0.026 | 0.0079 | -0.042 | -0.011 | 10.810 | 1 | 0.001 |
| WHO-FC I | Reference |  |  |  |  |  |  |
| WHO-FC II | 0.203 | 0.2220 | -0.232 | 0.638 | 0.835 | 1 | 0.361 |
| WHO-FC III | 0.468 | 0.2922 | -0.105 | 1.041 | 2.567 | 1 | 0.109 |
| WHO-FC IV | -0.275 | 0.5129 | -1.281 | 0.730 | 0.289 | 1 | 0.591 |
| ESC/ERS Risk I | Reference |  |  |  |  |  |  |
| ESC/ERS Risk II | 0.022 | 0.2067 | -0.383 | 0.427 | 0.012 | 1 | 0.914 |
| ESC/ERS Risk III | 0.250 | 0.2719 | -0.283 | 0.782 | 0.842 | 1 | 0.359 |
| ESC/ERS Risk IV | 1.463 | 0.5246 | 0.435 | 2.491 | 7.778 | 1 | 0.005 |
| Number of co-morbidities | 0.051 | 0.0413 | -0.030 | 0.132 | 1.540 | 1 | 0.215 |
| Disease duration (years) | 0.037 | 0.0135 | 0.010 | 0.063 | 7.370 | 1 | 0.007 |
| Occupation |  |  |  |  |  |  |  |
| Retired | Reference |  |  |  |  |  |  |
| Employed/Self-employed | -0.805 | 0.3055 | -1.404 | -0.206 | 6.938 | 1 | 0.008 |
| Disable | 0.252 | 0.2705 | -0.278 | 0.782 | 0.867 | 1 | 0.352 |
| Other | -0.034 | 0.3263 | -0.673 | 0.606 | 0.011 | 1 | 0.917 |

Table S8. Results of the generalized linear regression model including PAH subtype, WHO-FC, and ESC/ERS Risk.

| **Parameter** | **Coefficient** | **Std. Error** | **95% Wald Confidence Interval** | | **Hypothesis Test** | | |
| --- | --- | --- | --- | --- | --- | --- | --- |
|  |  |  | Lower | Upper | Wald Chi-Square | df | Sig. |
| **(Intercept)** | 11.700 | 0.323 | 11.066 | 12.334 | 1309.679 | 1 | <0.001 |
| **Female** | -0.052 | 0.126 | -0.299 | 0.196 | 0.169 | 1 | 0.681 |
| **Age** | -0.009 | 0.004 | -0.017 | 0.000 | 3.950 | 1 | 0.047 |
| **PAH subgroup 1.1** | Reference |  |  |  |  |  |  |
| **PAH subgroup 1.2** | -0.152 | 0.275 | -0.691 | 0.387 | 0.304 | 1 | 0.581 |
| **PAH subgroup 1.3** | -0.298 | 0.373 | -1.030 | 0.434 | 0.637 | 1 | 0.425 |
| **PAH subgroup 1.4** | -0.064 | 0.125 | -0.308 | 0.180 | 0.263 | 1 | 0.608 |
| **PAH subgroup 1.6** | -0.206 | 0.304 | -0.801 | 0.389 | 0.460 | 1 | 0.498 |
| **WHO-FC I** | Reference |  |  |  |  |  |  |
| **WHO-FC II** | 0.379 | 0.165 | 0.055 | 0.703 | 5.261 | 1 | 0.022 |
| **WHO-FC III** | 0.745 | 0.208 | 0.336 | 1.154 | 12.770 | 1 | <0.001 |
| **WHO-FC IV** | 0.970 | 0.460 | 0.068 | 1.872 | 4.447 | 1 | 0.035 |
| **ESC/ERS Risk I** | Reference |  |  |  |  |  |  |
| **ESC/ERS Risk II** | -0.226 | 0.155 | -0.530 | 0.078 | 2.125 | 1 | 0.145 |
| **ESC/ERS Risk III** | -0.339 | 0.208 | -0.747 | 0.069 | 2.646 | 1 | 0.104 |
| **ESC/ERS Risk IV** | 0.274 | 0.495 | -0.697 | 1.244 | 0.305 | 1 | 0.581 |
| **Treprostinil** | 1.432 | 0.175 | 1.089 | 1.774 | 67.135 | 1 | <0.001 |

Table S9. Results of the sensitivity analysis in which 4-week and 6 month outcomes were annualized using lower or higher multiplication factors.

|  | **All (N=124)** | **Lower estimate** | | | **Higher estimate** | | |
| --- | --- | --- | --- | --- | --- | --- | --- |
|  |  | **Mean** | **95% Confidence Interval** | | **Mean** | **95% Confidence Interval** | |
|  |  |  | **Lower** | **Upper** |  | **Lower** | **Upper** |
| Diagnostic costs | 2,359 | 1,887 | 1,645 | 2,130 | 2,831 | 2,467 | 3,194 |
| Outpatient visit to PAH centre costs | 1,491 | 1,193 | 972 | 1,414 | 1,790 | 1,458 | 2,121 |
| Treatment costs | 90,205 | 72,164 | 55,439 | 88,889 | 108,246 | 83,158 | 133,334 |
| Hospitalization costs | 9,288 | 7,430 | 2,016 | 12,844 | 11,145 | 3,024 | 19,266 |
| GP visit costs | 815 | 626 | 453 | 800 | 877 | 634 | 1,121 |
| Specialist visits costs | 3,063 | 2,356 | 1,560 | 3,152 | 3,298 | 2,184 | 4,412 |
| Formal care costs | 1,894 | 1,457 | 715 | 2,199 | 2,040 | 1,001 | 3,078 |
| **Total direct costs** | **109,114** | **87,114** | **69,043** | **105,184** | **130,227** | **103,161** | **157,292** |
| Workdays lost costs | 16,944 | 13,034 | 8,530 | 17,538 | 18,248 | 11,942 | 24,553 |
| Household chores costs | 7,469 | 5,745 | 3,194 | 8,297 | 8,044 | 4,471 | 11,616 |
| Informal care costs | 5,431 | 4,178 | 1,682 | 6,673 | 5,849 | 2,355 | 9,343 |
| **Total indirect costs** | **29,844** | **22,957** | **16,366** | **29,548** | **32,140** | **22,913** | **41,367** |
| **Total costs** | **138,959** | **110,071** | **89,703** | **130,439** | **162,367** | **132,179** | **192,554** |

Figure S1. Estimated total costs per patients per year, by gender (EUR).


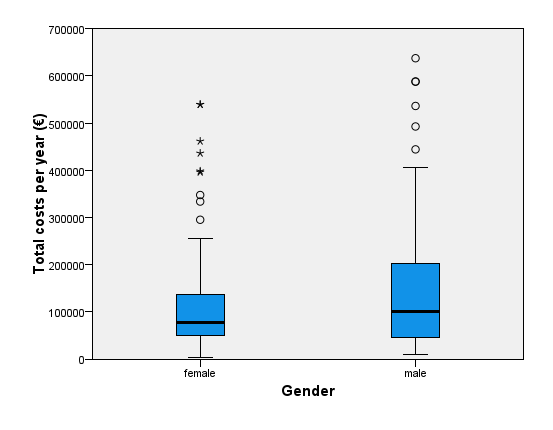


Figure S2. Estimated direct costs per patients per year, by gender (EUR).


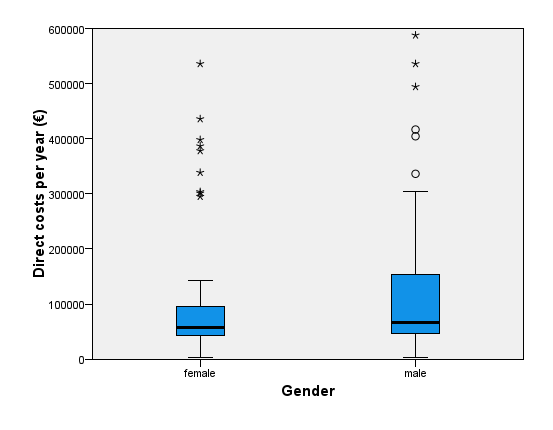


Figure S3. Estimated indirect costs per patients per year, by gender (EUR).


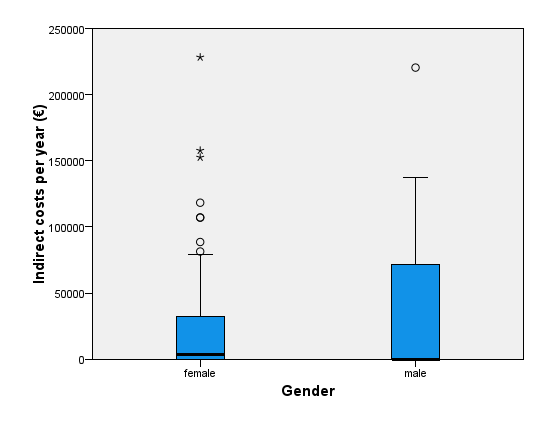


Figure S4. Estimated total costs per patients per year, by age group (EUR).


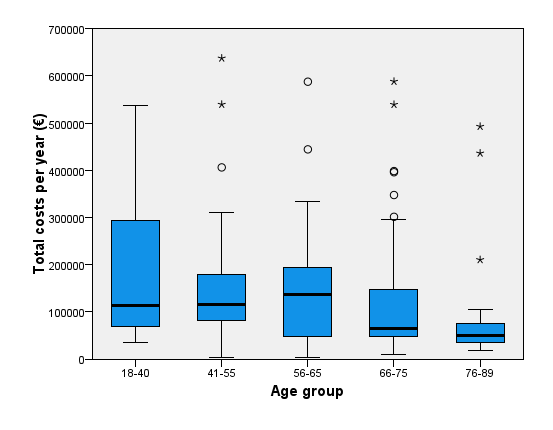


Figure S5. Estimated direct costs per patients per year, by age group (EUR).


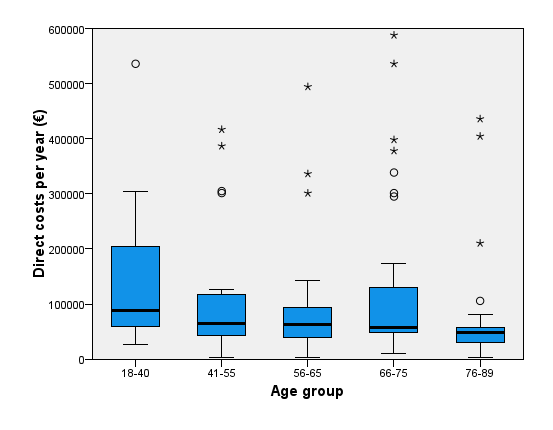


Figure S6. Estimated indirect costs per patients per year, by age group (EUR).

**
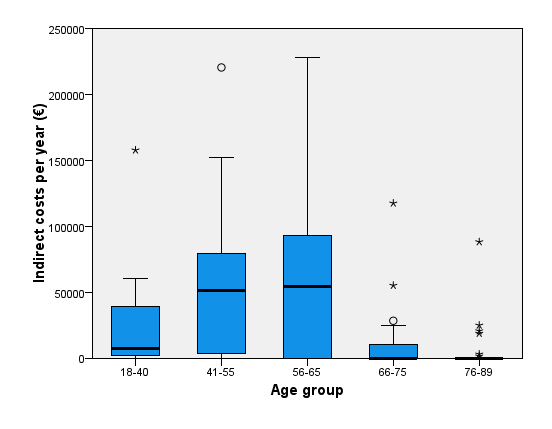
**

Figure S7. Estimated total costs per patients per year, by gender and WHO functional class (EUR).


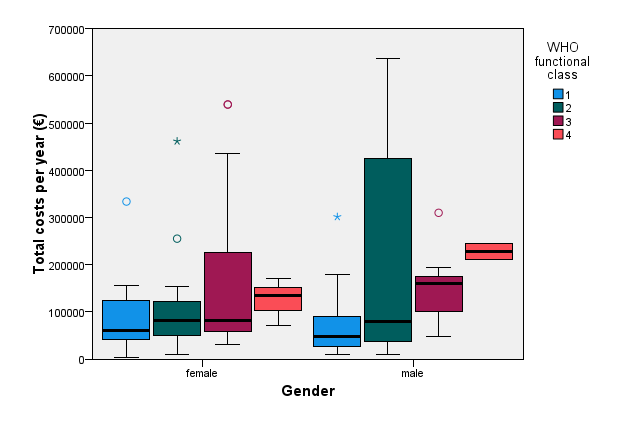


Figure S8. Estimated total costs per patients per year, by gender and ESC/ERS Risk (EUR).


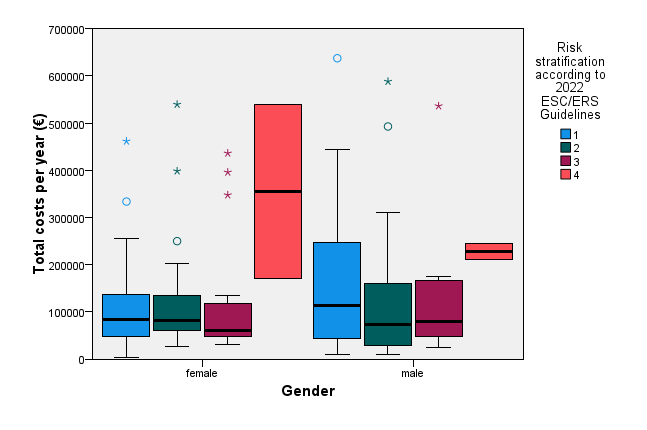


Figure S9. Estimated total costs per patients per year, by age group and WHO functional class


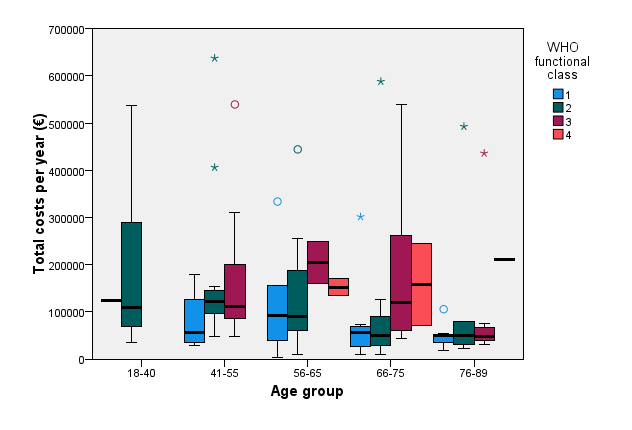


Figure S10. Estimated total costs per patients per year, by age group and ESC/ERS risk (EUR).


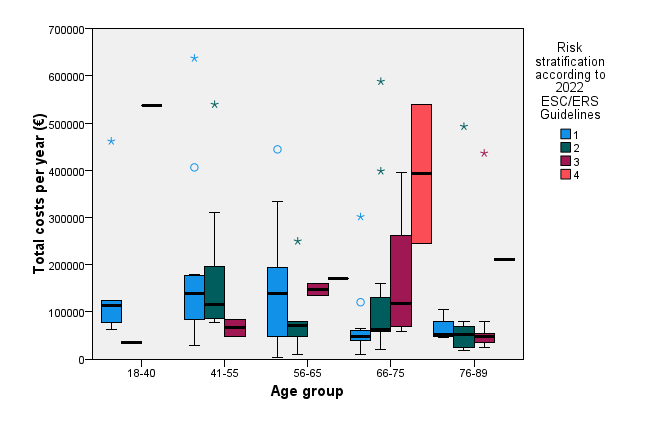


Figure S11. Estimated total costs per patients per year, by years since PAH diagnosis (EUR).


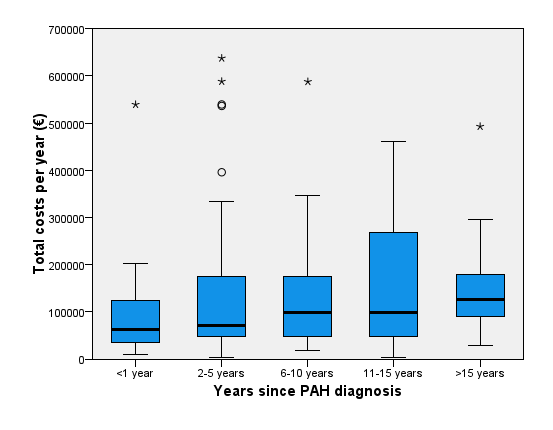


Figure S12. Estimated total costs per patients per year, by PAH subtype (EUR).


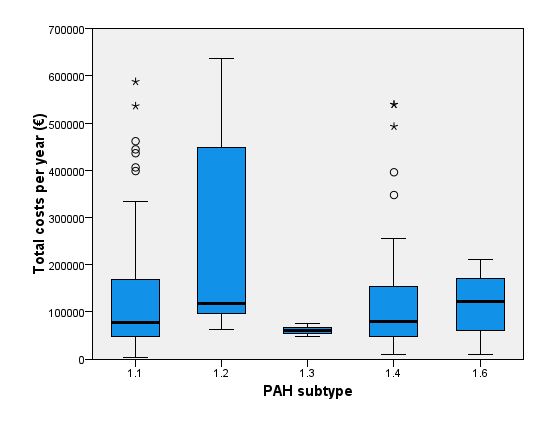


Figure S13. Estimated total costs per patients per year, by number of co-morbidities (EUR).


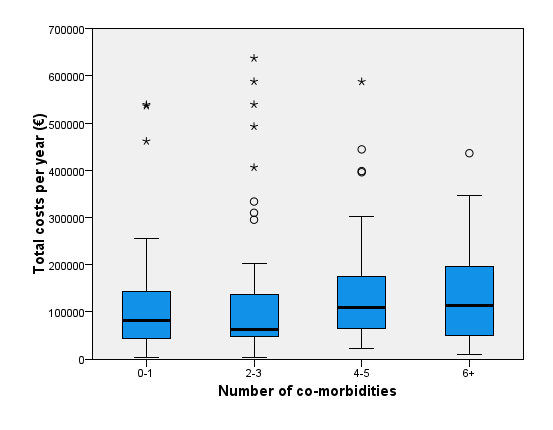


Figure S14. Estimated total costs per patients per year, by number of prescriber PAH drugs (EUR).


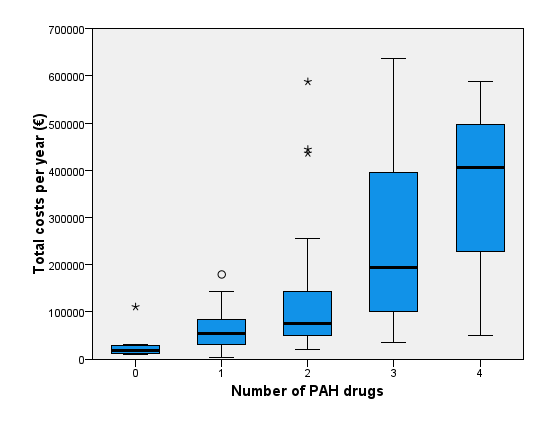


Figure S15. Estimated total costs per patients per year, by gender and years since PAH diagnosis (EUR).


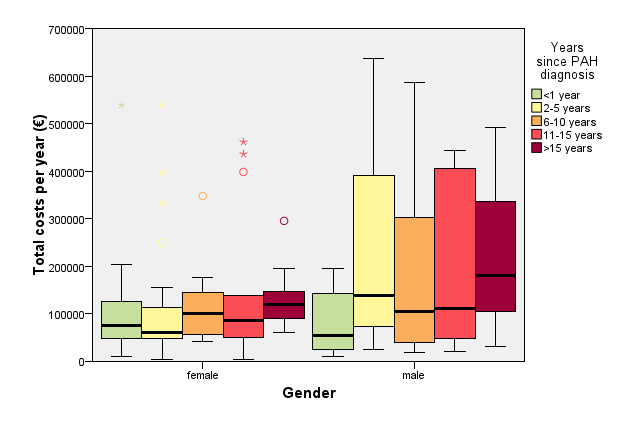

Supplement: S1 File — Additional tables and figures. (DOCX) [file pone.0348190.s001.docx]
